# timeClip: pathway analysis for time course data without replicates

**DOI:** 10.1186/1471-2105-15-S5-S3

**Published:** 2014-05-06

**Authors:** Paolo Martini, Gabriele Sales, Enrica Calura, Stefano Cagnin, Monica Chiogna, Chiara Romualdi

**Affiliations:** 1 Department of Biology, University of Padova, Padova, Italy; 2 Department of Statistics, University of Padova, Padova, Italy

## Abstract

**Background:**

Time-course gene expression experiments are useful tools for exploring biological processes. In this type of experiments, gene expression changes are monitored along time. Unfortunately, replication of time series is still costly and usually long time course do not have replicates. Many approaches have been proposed to deal with this data structure, but none of them in the field of pathway analysis. Pathway analyses have acquired great relevance for helping the interpretation of gene expression data. Several methods have been proposed to this aim: from the classical enrichment to the more complex topological analysis that gains power from the topology of the pathway. None of them were devised to identify temporal variations in time course data.

**Results:**

Here we present timeClip, a topology based pathway analysis specifically tailored to long time series without replicates. timeClip combines dimension reduction techniques and graph decomposition theory to explore and identify the portion of pathways that is most time-dependent. In the first step, timeClip selects the time-dependent pathways; in the second step, the most time dependent portions of these pathways are highlighted. We used timeClip on simulated data and on a benchmark dataset regarding mouse muscle regeneration model. Our approach shows good performance on different simulated settings. On the real dataset, we identify 76 time-dependent pathways, most of which known to be involved in the regeneration process. Focusing on the 'mTOR signaling pathway' we highlight the timing of key processes of the muscle regeneration: from the early pathway activation through growth factor signals to the late burst of protein production needed for the fiber regeneration.

**Conclusions:**

timeClip represents a new improvement in the field of time-dependent pathway analysis. It allows to isolate and dissect pathways characterized by time-dependent components. Furthermore, using timeClip on a mouse muscle regeneration dataset we were able to characterize the process of muscle fiber regeneration with its correct timing.

## Background

Time course gene expression experiments are widely used to study the dynamics of biological processes. Usually, the main goal of such experiments is to identify genes modulated along a biological process or after a system perturbation (such as drug treatments or genetic modifications). However, time course data are costly and usually long time series have few or no replicates. In this context a differentially expressed gene can be defined as a gene with the expression profile changing significantly along time and/or across multiple conditions. Several statistical models have been proposed to account for clusters and differential expression in the contest of time series with [1-18] and without replicates [10,19-21], but none of them were proposed in the context of pathway analysis. Pathway analysis has acquired great relevance in the last years especially for the ability to increase interpretability of gene expression results. Expression experiments typically provide lists of differentially expressed genes (DEGs) that represent the starting point for result interpretation. This step is not trivial and remains challenging for this type of analysis. The grouping of genes into functionally related entities (such as pathways) is of great help in the interpretation of the results. Several methods have been proposed to this aim, based on very different statistical tests and null hypotheses [22,23]. Broadly speaking, they can be divided into the classical enrichment analysis [24-28], working on gene lists selected through a gene-level test, and the novel global and multivariate approaches [29-37], that define a model for the whole gene set (see [22,38-40] for a comprehensive reviews and comparative analysis). The latter can be further divided into 'topological' and 'non-topological' methods according to their ability to gain power from the topology of the pathway [25,35,36,41-43].

A pathway is a complex structure comprising chemical compounds mediating interactions and different types of gene groups (e.g. protein complexes or gene families) that are usually represented as single nodes but whose measures are not available using gene expression data. However, after appropriate biologically-driven conversion [44,45], a biological pathway can be represented as a graph where genes and their interactions are, respectively, nodes and edges of the graph.

Taking advantage of the structure of the graph, Massa *et al*. [35] used Gaussian graphical model theory to test both differences in mean and in covariance matrices between two experimental conditions. In particular, graphical models are useful to decompose the overall graph (obtained from a pathway) into smaller components (cliques), that can be explored and tested in detail. Martini *et al*. [36] proposed an extension of this method, called CliPPER, based on a two-step empirical approach. In the first step, it selects pathways with covariance matrices and/or means significantly different between experimental conditions dealing with the *p >> n *case; in the second step, it identifies the sub-paths (called signal paths) most associated with the phenotype.

Pathway analysis is mainly tailored to two-groups comparisons and few efforts have been dedicated to the time course design. Here, we propose a modification of [36], called timeClip, to deal with long time course data without replicates. Specifically, timeClip combines principal component analysis, regression models and graph decomposition to explore temporal variations across and within pathways. Moreover, timeClip implements an easy and effective visualization of the dynamics of the pathways.

On simulated datasets, timeClip shows good performances in term of power, specificity and sensitivity. Using real data on mouse muscle regeneration [46], we obtain excellent results in agreement with the scientific literature.

## Method

### Pathway annotation

A critical step in the field of topology based pathway analyses is the availability and the quality of the pathway topology. Our group has recently developed graphite a Bioconductor package for the storage, interpretation and conversion of pathway topology to gene-only networks [44]. graphite discriminates between different types of biological gene groups and propagates gene connections through chemical compounds. Specifically, protein complexes are expanded into a clique (all proteins connected to the others), while the gene families are expanded without connections among them; see [44,45] for more details. The current version of graphite Bioconductor package is limited to human, so here we build a dedicated graphite package for mouse KEGG pathways. This package is available at http://romualdi.bio.unipd.it/wp-uploads/2013/10/graphite.mmusculus_0.99.2.tar.gz.

### timeClip: general approach

**Figure 1:**
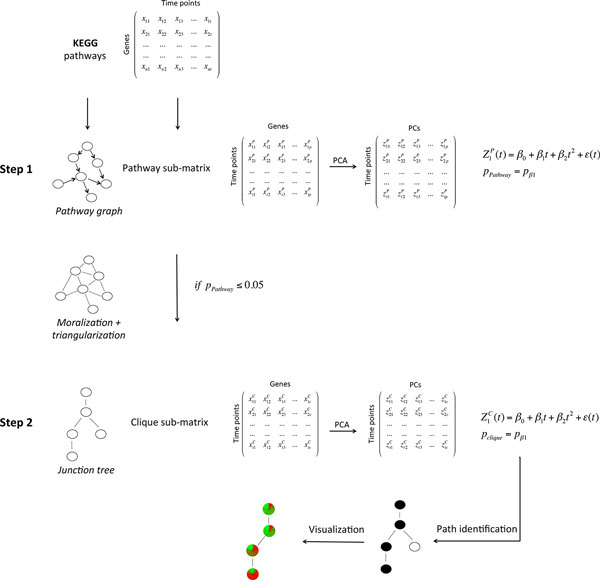
**timeClip**. Global overview of timeClip approach.

A pathway is composed by multiple genes so to reduce the dimension of a whole or of a portion of a pathway, we used principal component analysis. Then the first principal component is explored for temporal variation. A vast amount of techniques exist for analyzing regularly sampled time series. Unfortunately, the irregular sampling of the values (a common practice in biology) makes direct use of such estimation techniques impossible. To avoid the well known biases associated with the most common approach for irregularly sampled time series based on transforming unevenly-spaced data into equally spaced observations using some form of interpolation, here we propose to use a regression model combining a polynomial trend and a continuous-time Gaussian autoregressive process of order 1 (AR(1)). Then, timeClip resembles the two-steps approach of CliPPER. In the first step, the whole pathway is explored for its temporal variation. If the pathway is defined as time-dependent, in the second step, timeClip decomposes the pathway into a junction tree and highlights the portion mostly dependent on time. A general schema of the approach is summarized in Figure 1.

#### Step 1: exploring the whole pathway

Let *X_n × t _*be the normalized log transformed gene expression matrix with genes on the rows and experiments (equal to time points t) on the columns. Let $X_{p\times t}^{P}$ the sub-matrix of genes belonging to pathway *P*. Pathway *P *has *p *genes. Then, on the transpose of *X^P^, X^P'^*, we perform principal component analysis (PCA). We used both the classical (R package stats) and the robust (rrcov R package) version of PCA. Let $Z_{p\times t}^{P}$ be the scores matrix and $L_{p\times t}^{P}$ the loadings matrix. We call $Z_{1}^{P},\cdots \;,Z_{p}^{P}$ the *p *principal components. In this way, the first PCs summarize the temporal variation of the genes in pathway *P *(if present). Thus, from now on we will indicate $Z_{i}^{P}$ as $Z_{i}^{P}\left(t\right)$. A similar approach was recently proposed by [15] (PCA-maSigFun). PCA-maSigFun uses principal component analysis to identify temporally-homogeneous groups of gene within the pathway.

Then, for irregularly sampled time series we assume that our irregularly sampled signal $Z_{i}^{P}\left(t\right)$ can be decomposed as *Z*(*t*) = *p*(*t*) + ∈(*t*), where *p*(*t*) is a deterministic function, hereafter called "trend", and *∈*(*t*) is the realization of a stationary stochastic process with mean zero. Extensive exploratory analysis suggests that a reasonable choice for the trend component is a polynomial of degree 2 in *t*, i.e.,

$$p\left(t\right)=\beta_{0}+\beta_{1}t+\beta_{2}t^{2}$$

with *β*_1 _capturing existing temporal behaviors of $Z_{1}^{P}\left(t\right)$ and *β*_2 _correcting for potential non linearities.

Moreover, we assume that ∈(*t*) follows a continuous-time Gaussian autoregressive process of order 1. The model is fitted using generalized least squared (as implemented in nlme R package). The representative *p *- *value *of pathway P, *p_P_*, is then taken to be the *p *- *value *of the test of nullity of *β*_1 _(obtained by a t-test as implemented in the gls function of the nlme R package). Bonferroni correction is used to adjust *p *- *values *for multiple tests.

We evaluated the possibility to fit a polynomial regression not only on the first PC, but also on few additional $Z_{i}^{P}$, with *i *= 2, 3. However, we did not find significant improvements in the final list of significant time-dependent pathways (data not shown).

#### Step 2: decomposing the pathway

Pathways declared as time-dependent in step 1 are then moralized, triangulated and decomposed into a junction tree as described in [36].

Briefly, moralization inserts an undirected edge between two nodes that have a child in common and then eliminates directions on the edges; triangulation inserts edges in the moralized graph so that in the moralized graph all cycles of size ≥ 4 have chords, where a chord is defined as an edge connecting two non-adjacent nodes of a cycle. A clique in the triangulated graph is a complete subgraphs having all its vertices joined by an edge while a junction tree construction is a hyper-tree having cliques as nodes and satisfying the running intersection property according to which, for any cliques *C*_1 _and *C*_2 _in the tree, every clique on the path connecting *C*_1 _and *C*_2 _contains *C*_1 _∩ *C*_2 _[36,47]. For a given graph there could be more than one junction tree. Here we force the root of the junction tree to be in agreement with the structure of the pathway.

A clique *k *of pathway *P*, noted as $C_{k}^{P}$ (with *k *= 1,..., *K*), is composed by a subset of genes in *P*, $c_{k}^{P}$. Let $X_{ck}^{p}$ be the sub-matrix of *X *corresponding to the genes of the clique $C_{k}^{P}$. For each clique *k *of *P *we apply the same approach as described in step 1: PCA transformation and then a linear model with polynomial trend and autoregressive process of order 1 on the first PCs. The *p *- *value *of clique *k *in pathway *P*, $p_{C_{k}^{P}}$ is given by the *p *of the *β*_1 _of the polynomial regression. Finally, the best time-dependent paths within a pathway P, hereafter called $S_{P_{j}}$, *j = 1,..., J*, are identified using the relevance measure as described in [36]. Briefly, a path is a chain of consecutive time-dependent cliques $\left(p_{C_{k}^{P}}\leq 0.05\right)$ with gaps at most of size one. Then, for each path in the pathway a cumulative score is calculated along the path: lower the the *p *-- *value *of a clique in the path, higher the contribution to the score, in case of gap the score is penalized. The final score of a path is the maximum value reached by the score along the path. Then, the score is normalized for the path length; this quantity is called relevance [36].

As final results, for each time-dependent pathway, we report a list of relevant paths, ranked according to their relevance. Currently, step 2 is the most innovative feature of timeClip and, as far as we known, there are no existing tools using a similar strategy.

### Simulated data

As some paths may be declared time-dependent by timeClip step 2 simply as a consequence of type I errors in timeClip step 1, we used a simulation to evaluate the percentage of false positives under the null hypothesis and to estimate the statistical power in different scenarios.

#### False positive rate estimation

Given a pathway *P *and its graph structure (G), for 1,000 runs we randomly generate a gene expression matrix *X*_*n *× *t *_from a multivariate normal distribution with zero mean and variance ∑, with $\sum \in S^{+}\left(G\right)$ (where *S*^+^(*G*) is the set of symmetric positive definite matrices with null elements corresponding to the missing edges of G). In this case, gene expression profiles are time independent. Then, for each run we calculate *p_P _*(either for the case of irregularly and regularly sampled time points, see Section Step 1: exploring the whole pathway). Under this scenario, at the nominal level *α *= 0.05 we expect a number of rejections around 5%. We repeat the simulation for different values of *n *(*n *= 5, 10, 15, 20, 25, 30) and *t *(*t *= 5, 10, 15, 20, 30).

#### Power estimation

In order to be sure that the model were able to identify time-dependency coming from different models, we simulate data using polynomial models, autoregressive models of order 1 and a combination of both (polynomial models with autocorrelated errors). Then, the power is estimated for irregularly and regularly sampled time points.

Given a pathway *P *and its graph structure (G), for 1,000 runs we randomly generate a gene expression matrix *X*_(*n *− *s*) × *t *_from a multivariate normal distribution with zero mean and variance ∑ with $\sum \in S^{+}\left(G\right)$. Then, the expression profiles of the remaining *s *genes, with *s *≤ *n *are simulated to have different degree of time-dependency. Specifically, we use polynomial models (Equation 1), autoregressive models of order 1 (Equation 2, where ∈* is a white noise) and the combination of both (Equation 3, where *∈ *an AR(1)).

(1) $$x_{s}\left(t\right)=\alpha_{0}+\alpha_{1}t+\alpha_{2}t^{2}+\epsilon^{*}$$

(2) $$x_{s}\left(t\right)=\varphi_{0}+\varphi_{1}x_{s}\left(t-1\right)+\epsilon^{*}$$

(3) $$x_{s}\left(t\right)=\alpha_{0}+\alpha_{1}t+\alpha_{2}t^{2}+\varphi_{1}\epsilon_{s}\left(t-1\right)$$

The coefficients α* are independently generated from a U(−5, 5), and *φi *are generated so as to achieve stationarity. In this way, we simulate expression profiles with different degrees of temporal variations. Then, for each run we calculate *p_P _*(see Section Step 1: exploring the whole pathway). Under this scenario, the number of rejection estimates the statistical power. We repeat the simulation for different combinations of *φ, n, s *and *t*.

### Real data: muscle regeneration model

The benchmark dataset used [46] (GSE469) follows mouse muscle regeneration after intra-muscluar injection of cardiotoxin. Regeneration process is followed for 27 unevenly spaced time-points with only two technical replicates for each time-point. Expression data were produced using single channel Affimetrix microarrays. The probes in the platform were annotated with EntrezGene custom CDF (version 14) [48] and data was normalized using the robust multi array analysis (rma) and quantile normalization. Then, technical replicates were averaged to get one measure for every time-point.

### Implementation and visualization: the wheel of time

timeClip is implemented as an R package available from the authors. The package allows to analyze equally and non-equally spaced time series according to the user setting. To get better insights into the temporal activation of the different portions of the pathway, we develop a new way of visualization using Cytoscape software [49] and Rcytoscape Bioconductor package. The visualization, called the wheel of time, allows visualizing pie charts inside network nodes. For each pathway, timeClip exports in Cytoscape the structure of the junction tree where each time-dependent clique has a pie chart that represents the time trend. Specifically, the pie is divided into as many slices as the number of time points in the dataset. Each slice in the pie is colored (from green to red) according to the scores of first principal component: the higher the value, the stronger the activation of a clique in a specific time point (red color) and viceversa (green).

## Results and discussion

Many biological processes need to be followed and monitored along time. In these cases time course designs are ideals: higher the number of time points, finest the monitoring process. However, long time courses are often characterized by small or no replicates. Here, we present timeClip, a two-step approach to perform topological pathway analysis for time course gene expression data, specifically tailored to long time series without replicates (Figure 1). In the first step, we select pathways that show time dependency. In the second step, the selected pathways are decomposed into cliques and the time-dependent portions are isolated. In the next sections, we will show the performance of timeClip using simulated and real datasets.

### Simulations results

Two simulation strategies have been considered. The first one was designed to estimate the number of false positives under the null hypothesis of no temporal variation, the second to estimate the statistical power (see section method for details).

Table 1 and Table S1 (Additional file 1) report the percentage of false positives obtained with different *n *and *t *for the irregularly and regularly sampled time points, respectively. The average false positive percentage for each *t *and *n *is always limited to ~4-5%, with the exception of small time series (*t *= 5) and equally spaced time points where it is slightly higher. Thus, we can conclude that, in general, for long time series we have an excellent control of type I error even with exceptionally low sample sizes.

**Table 1:** Simulation results - False positives rate with different pathway dimensions *n *and irregularly sampled time points *t*.

	*t *= 5	*t *= 10	*t *= 15	*t *= 20	*t *= 25	*t *= 30
*n *= 5	0.04	0.03	0.03	0.05	0.04	0.04
*n *= 10	0.04	0.04	0.04	0.04	0.04	0.04
*n *= 15	0.03	0.03	0.03	0.03	0.04	0.04
*n *= 20	0.04	0.03	0.05	0.04	0.03	0.04
*n *= 25	0.04	0.04	0.04	0.04	0.04	0.04
*n *= 30	0.04	0.04	0.04	0.05	0.03	0.04

Table 2 and Table S2 (Additional file 1) report the number of true positives obtained with *n *= 30 and different *t *and *s *for equally and not-equally spaced time points respectively. Here, the genes with temporal variation are simulated using different models (if *s *is the number of time-dependent genes among the *n *of the pathway, we simulate *s*/3 with polynomial, *s*/3 with AR(1) and *s*/3 with the combination of both). As expected, the power increases with the increase of *t *and *s*: the longer the time course and the higher the number of time dependent genes s within the pathway, the higher the power.

**Table 2:** Simulation results - Power estimate in case of *n ***= 30 **and different time course length *t *and time dependent genes *s*.

	*s *= 3	*s *= 6	*s *= 9	*s *= 21	*s *= 15	*s *= 30
*t *= 5	0.08	0.08	0.09	0.1	0.1	0.09
*t *= 10	0.53	0.47	0.47	0.47	0.44	0.45
*t *= 15	0.68	0.61	0.55	0.53	0.48	0.49
*t *= 20	0.74	0.67	0.67	0.62	0.61	0.59
*t *= 25	0.75	0.68	0.65	0.63	0.61	0.64
*t *= 30	0.84	0.77	0.82	0.84	0.81	0.84

Irregularly sampled time points.

Specifically, when the time course is short (*t *= 10 − 20) the maximum power reaches 60%, while with long time series *t *= 30 the power is above 80%. Moreover, it is worth noting that the increase of the time dependent genes does not affect significantly the power level. The greater impact that the number of time points has on statistical power with respect to the number of time-depending genes can be explained by the presence of two steps in our strategy: i) a data reduction step (with PCA on genes within pathways) and ii) a model-fitting step of the reduced variables on time points. PCA is an efficient method to detect variance components in the data. Thus, even in case of a small number of time-dependent genes, the first PC is able to capture the time trend when present. On the other hand, once the trend is captured, the goodness of fit of the regression model increases by increasing the number of time points. The use of robust PCA does not change the performance of the method substantially (data not shown).

### Case study: muscle regeneration model

#### Step 1 results

In step 1 every pathway is explored for its temporal dependence. In the benchmark dataset, we have to deal with 27 not equally spaced times (14 of which are equally spaced).

**Figure 2:**
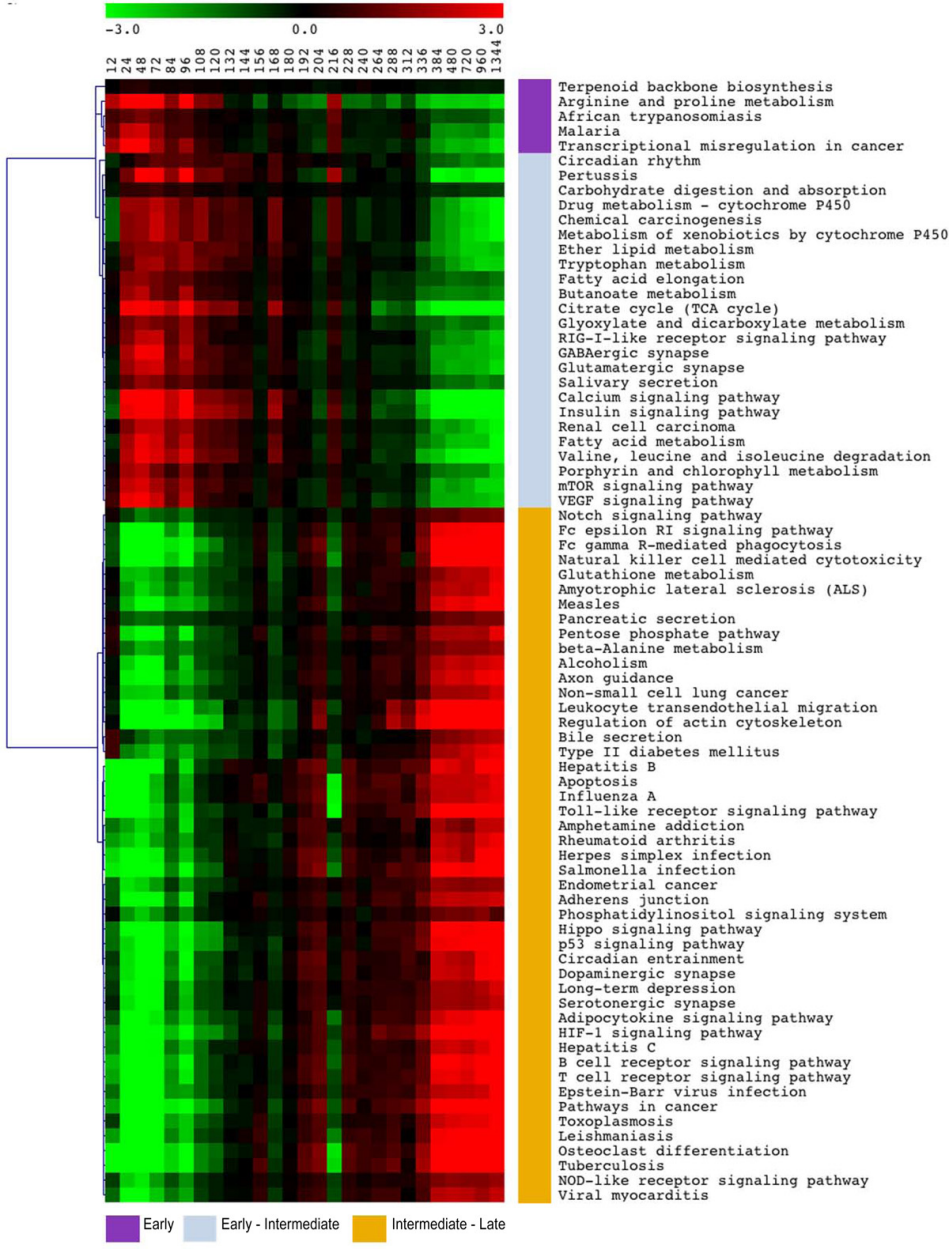
**Heat map of pathway PCs**. Heat map colored according to the expression of the first PCs from green to red. According to the color pattern, pathways are divided in early, early-intermediate and late-intermediate. Time is measured in hours after treatment.

Comparing step 1 results for equally and not equally spaced time-point we obtain an overlap of 70%. This high degree of overlap makes us confident about the reliability of our approach. We summarized the results in the heat map of Figure 2 (values reported in Additional file 2). The heat map is obtained using the scores of the first principal component of each time-dependent pathway. From the unsupervised cluster analysis, we can define 3 pathway groups characterized respectively by a 'very early','early-intermediate' and 'intermediate-late' activation. Pathways characterized by a very early activation like 'Malaria' and 'African trypanosomiasis' reflect the early activation of the inflammation processes deputed to clean injured fibers. These processes are carried-out by macrophages that have a central role in the 'Malaria' and 'Africa trypanosomiasis' pathways. Macrophages clean up injured fiber and release growth factors like vascular endothelial growth factor (VEGF) and hepatocyte growth factor (HGF) [50].

In the early-intermediate pathway group, we can see the effects of the early signal secretion: in fact, the group contains pathways like 'mTOR signaling pathway', 'VEGF signaling pathway', 'Insulin signaling pathway' and other metabolic pathways like 'Ether lipid metabolism' and 'Citrate cycle (TCA cycle)'. Globally, these pathways indicate that the regeneration progress has begun.

'mTOR signaling pathway', probably the most important pathway in the muscle regeneration, on one side sustains VEGF signaling and on the other promotes protein production needed for clonal expansion of the myoblasts, their growth and fusion. In particular, mTOR integrates growth factor signaling with a variety of signals from nutrients (amino acids metabolism activate mTOR pathway) and cellular energy status [51]. The energy status of the cell is indeed monitored by those pathways involved in energy metabolism like 'carbohydrate digestion and adsorption', 'Citrate cycle (TCA cycle)' and 'Fatty acid metabolism'. These processes are very important in the regeneration process, in fact, it was demonstrated that glycolitic metabolism is restored after three days from myofibril formation [52].

Intermediate-late activation pathways mainly present pathways involved in inflammatory responses like 'B and T Cell receptor signaling pathway', 'Toll-like receptor signaling pathway', 'Adipocytokine signaling pathway' and 'Leukocyte transendothelial migration'. Recent discoveries reveal complex interactions between skeletal muscle and the immune system that regulate all phases of the muscle regeneration [50]. Moreover in this pathway group there is the 'Axon guidance' and 'Dopaminergic synapse' pathways that are involved in nervous impulse transduction. We can speculate that at the end of the regenerative processes nervous system can contact the restored contractile cells to ensure and maintain their functionality.

This contains also pathways involved in signaling transduction like 'HIF-1 signaling pathway'. HIF-1 has been recently demonstrated to be essential for skeletal muscle regeneration in mice [53]. In fact this pathway manages a plethora of signals and interface with pathways like mTOR signaling pathway, PI3K-Akt signaling pathway, MAPK signaling pathway, Citrate cycle (TCA scycle), Calcium signaling pathway, VEGF signaling pathway and Ubiquitin mediated proteolysis. Together with all these pathways, 'HIF-1 signaling pathway' finely tune the balance between oxygen consumption.

In step 1, we are able to see only the strongest signals and not always the pathway name alone reflects the activity of the pathway. To tackle the complexity of the pathway, timeClip step 2 deeply investigates the timing activation of different portion of the pathway.

#### Step 2 results

In the second step, we focused on the the Akt-mammalian target of rapamycin (mTOR) signaling pathway. It regulates a pletora of signals: cell growth, VEGF signaling pathway, autophagy and its action is related to other pathways known to be involved in the muscle regeneration like Insulin signaling pathway and MAPK signaling pathway [54].

**Figure 3:**
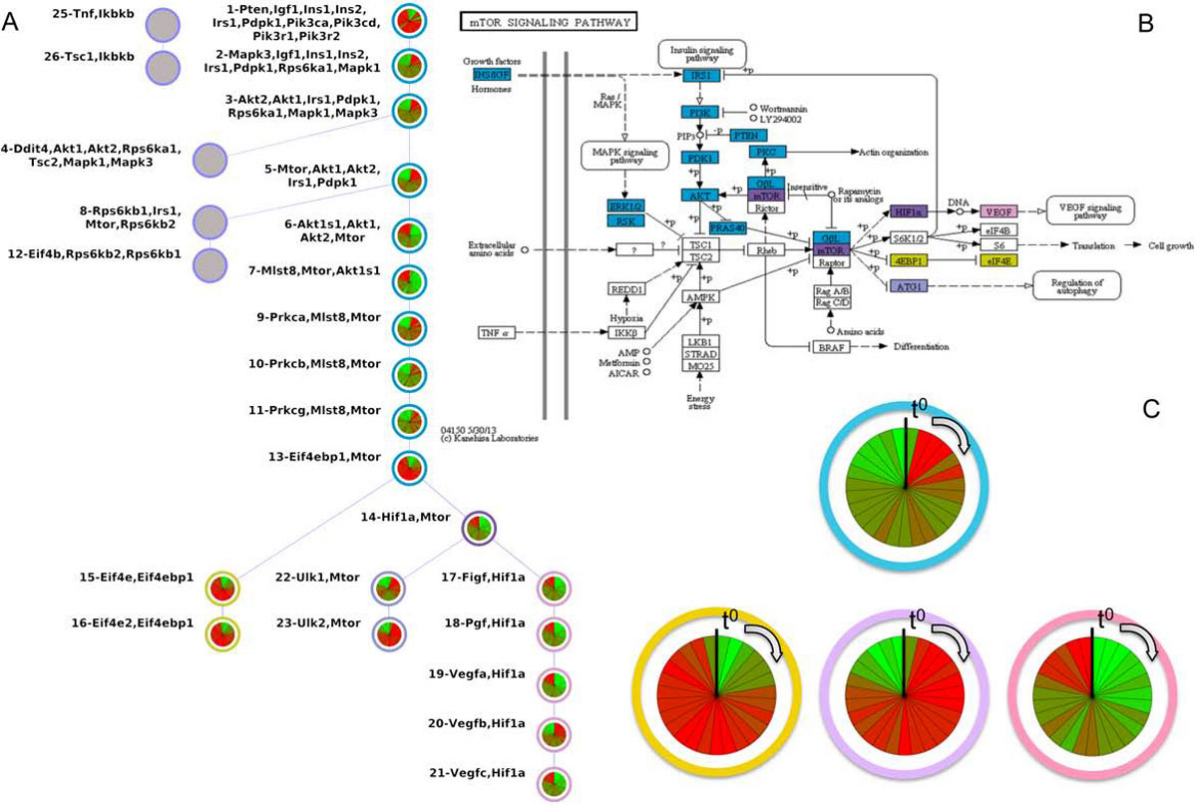
**Activation of the mTOR signaling pathway**. Panel A. Junction tree of the mTOR signaling pathway (using graphite R package and database KEGG). The top ranked time-dependent paths identified in timeClip step 2 are highlighted using the wheel of time visualization. Panel B. KEGG representation of mTOR signaling pathway. Genes are colored according to the paths in panel A. Panel C. Enlargement of the wheels of time representative of the main block of mTOR signaling pathway: from *t*_0 _to *t*_27 _(clock-wise) every slice of the pie is colored according to the value of the clique first PC (green means no activation; red means activation).

The junction tree of mTOR signaling pathway (Figure 3A) starts with Igf1 (Insulin-like growth factor 1) as represented in the KEGG map (Figure 3B). Within mTOR signaling pathway we identified a total of 6 paths, ranked by their relevance score (Table 3).

**Table 3:** mTOR signaling pathway: relevant paths identified by timeClip step 2

path	starting clique	ending clique	lenght	Relevance	average Relevance
1;21	1	21	16	102.11	6.38
1;23	1	23	13	74.39	5.72
1;16	1	16	12	67.79	5.65
1;12	1	12	5	27.43	5.49
1;4	1	4	4	26.01	6.5
25;26	25	26	1	1.9	1.9

The most relevant of these paths goes from the 1*^st ^*to the 21*^st ^*clique and contains 16 cliques. The second and the third path share a big portion with the first one. This big portion goes from clique 1 to cliques 13 (blue nodes on the junction tree - Figure 3A) and contains genes like Igf1, Insulin, Mapk3, Mtor and Akt that globally represent the backbone of the pathway where the starting activating signal is regulated by Igf1. Then Pi3k, Mapk and Akt translate the signal and activate Mtor that organize the effectors. From the junction tree we can identify three different terminal effectors: the first, in pink, is the portion that brings to the VEGF signaling pathway. The second, in purple, is the regulation of autophagy and the third, in yellow, is the regulation of protein synthesis that is necessary for the skeletal muscle mass recovery during regenerating processes [55]. In the panel C of Figure 3, we summarized the timing of the 'mTOR signaling pathway' activation. With the wheel of time, we can see that the pathway backbone is activated in the early phases. The portion that brings to the VEGF signaling pathway is activated in the late phases. The effectors that bring to authophagy are switched off at the end of the regenerative precess while the activation of the protein synthesis begun from the early-intermediate phases and last till the end of the process.

Recently, as discussed before, it was demonstrated the involvement of HIF-1 in the skeletal muscle regeneration process [53]. We observed that the most relevant path of HIF-1 signaling pathway is 37 cliques long underlining its importance in this process. This path is activated by different growth factors (Igf, Ins, Egf) and signals are translated through Akt and mTOR towards HIF-1*α*/*β*. Hif-1*α *regulated many processes from the oxygen balance to apoptosis (See Additional file 3). Such downstream effectors confirm its importance in skeletal muscle regeneration in accordance with results obtained from [53].

## Comparison with other methods

In this section we compare timeClip step 1 results with the methods proposed by [15]. Step 2, that is the most innovative feature of timeClip, cannot be compared to any existing tool. [15] proposed two different strategies. The first one, called maSigFun, considers individual genes as different observations of the expression profile of the pathway. The second approach PCA-maSigFun uses PCA to identify groups of genes showing different time-dependencies. maSigFun did not give any significant time-dependent pathway using our dataset describing skeletal muscle regeneration (*p *≤ 0.05), while PCA-maSigFun returned 59 significant KEGG pathways (*p *≤ 0.05). 26 out of 59 (44%) pathways are in common with timeClip step 1 results. Indeed, both the methods retrieve mTOR signaling pathway, however PCA-maSigFun did not call HIF signaling pathway as significant, although it seems to be closely related to the muscle regeneration [53]. Most of the PCA-maSigFun specific pathways (15 out of 33) referred to metabolic processes like Inositol phosphate methabolism, Pyruvate metabolism, Tyrosine metabolism, Glycerolipid metabolism. The remaining pathways are highly heterogeneous and comprise Acute myeloid leukemia, Bladder cancer, Melanoma, Pancreatic cancer.

## Conclusions

Pathway analysis is a useful and widely used statistical approach to test groups of genes between two or more biological conditions. Although many efforts have been dedicated to implement novel gene set analysis in a multivariate and topological contexts, few of them deal with time course experiments. Time course experiments are used to monitor the dynamics of biological processes under physiological conditions or after perturbations.

In this context there is a clear trade-off between the number of time points and the number of replicates. In general, if the goal of the study is the identification of time-dependency, long time course are required at the expense of replicates; on the other hand, if the goal is the characterization of short term response a large number of replicates for each time point is required to increase statistical power. In general, there are few long time series datasets and in our opinion this is partly due to the experimental costs but also to the lack of effective methods to study and interpret results. Here, we present timeClip, an empirical two-step approach specifically tailored to long time course gene expression data without replicates. Using simulated data timeClip shows good performance in terms of controlling type I error and power. Furthermore, we successfully identify most of the key pathways involved in the early, middle and late phases of the skeletal muscle regeneration process. A visualization tool has also been implemented to tackle the dynamics of the transcriptome.

## Competing interests

The authors declare that they have no competing interests.

## Authors' contributions

CR define the concept proposed. PM developed the proposed methods and performed the analysis on gene expression data. GS and EC developed the computational infrastucture for pathway topology retrieval. CR and MC supervised the work and wrote the paper. SC participated in the biological discussion of the results. All Authors read and approved the final manuscript.

## Supplementary Material

Additional file 1 **Additional tables. This file contains additional tables mention on the text (pdf format)**. Click here for file

Additional file 2 **Figure 2 Heat map values. This file contains the values used to create the heat map in Figure 2. In column 2 the pathways that are called significant also by ****PCA-maSigFun** with an alpha <= 0.05 are marked with "*". In addition *p - values *and adjusted *p *- *values *(Bonferroni) for **timeClip**** are show in col 3 and 4 (tab delimited format)**. Click here for file

Additional file 3 **Activation of the HIF-1 signaling pathway. KEGG representation of HIF-1 signaling pathway. Genes of the 37 clique long path colored in cyan**. Click here for file

## Declarations

Pubblication of this article was founded by the University of Padova and CARIPARO Foundation.

This article has been published as part of *BMC Bioinformatics *Volume 15 Supplement 5, 2014: Italian Society of Bioinformatics (BITS): Annual Meeting 2013. The full contents of the supplement are available online at http://www.biomedcentral.com/bmcbioinformatics/supplements/15/S5

## References

[B1] Park T Yi S Lee S Lee S Yoo D Ahn J Lee Y Statistical tests for identifying differentially expressed genes in time course microarray experiments Bioinformatics 2003 19 694 703 12691981 10.1093/bioinformatics/btg068

[B2] Smyth G Limma: linear models for microarray data Bioinformatics and computational biology solutions using R and Bioconductor 2005 12837 12842

[B3] Tai Y Speed T A multivariate empirical Bayes statistic for replicated microarray time course data Ann Stat 2006 34 2387 2412

[B4] Yuan M Kendziorski C Hidden Markov Models for Microarray Time Course Data in Multiple Biological Conditions J Am Stat Assoc 2006 101 476 1323 1332

[B5] Sun W Wei Z Multiple Testing for Pattern Identification, With Applications to Microarray Time-Course Experiments J Am Stat Assocsss 2011 106 73 88

[B6] Ramsay J Silverman B Functional data analysis 2005 2005

[B7] Coffey N Hinde J Analysing time-course microarray data using functional data analysis - A review BMC Bioinf 2011 10 23

[B8] Xu X Olson J Zhao L A regression-based method to identify differentially expressed genes in microarray time course studies and its application in an inducible Huntington's disease transgenic model Human Mol Genet 2002 11 17 1977 1985 12165559 10.1093/hmg/11.17.1977

[B9] Bar-Joseph Z Gerber G Simon I Gifford D Jaakkola T Comparing the continuous representation of time-series expression profiles to identify differentially expressed genes Proc Nat Acad Sci USA 2003 100 10146 10151 12934016 10.1073/pnas.1732547100 PMC193530

[B10] Storey J Xiao W Leek J Tompkins R Davis R Significance analysis of time course microarray experiments Proc National Acad Sci USA 2005 102 36 12837 12842 10.1073/pnas.0504609102 PMC1201697 16141318

[B11] Hong F Li H Functional hierarchical models for identifying genes with different time-course expression profiles Biometrics 2006 62 534 544 16918918 10.1111/j.1541-0420.2005.00505.x

[B12] Liu X Yang M Identifying temporally differentially expressed genes through functional principal component analysis Biostatistics 2009 10 667 679 19602570 10.1093/biostatistics/kxp022

[B13] Chen K Wang J Identifying differentially expressed genes for time-course microarray data through functional data analysis Stat Biosci 2010 2 95 119

[B14] Ma P Zhong W Liu J Identifying differentially expressed genes in time course microarray data Stat Biosci 2009 1 144 159

[B15] Nueda M Sebastian P Tarazona S Garcia-Garcia F Dopazo J Ferrer A Conesa A Functional assessment of time course microarray data BMC Bioinformatics 2009 10 Suppl 6 S9 http://www.biomedcentral.com/1471-2105/10/S6/S9 19534758 10.1186/1471-2105-10-S6-S9 PMC2697656

[B16] Schliep A Costa IG Steinhoff C Sch?nhuth A Analyzing Gene Expression Time-Courses IEEE/ACM Transactions on Computational Biology and Bioinformatics 2005 2 3 179 193 17044182 10.1109/TCBB.2005.31

[B17] Ramoni MF Sebastiani P Kohane IS Cluster analysis of gene expression dynamics Proceedings of the National Academy of Sciences 2002 99 14 9121 9126 10.1073/pnas.132656399 PMC123104 12082179

[B18] Son YS Baek J A modified correlation coefficient based similarity measure for clustering time-course gene expression data Pattern Recogn Lett 2008 29 3 232 242

[B19] Han X Sung W Feng L Identifying differentially expressed genes in time-course microarray experiment without replicate J Bioinf Comput Biol 2007 5 281 296 10.1142/s0219720007002655 17589962

[B20] Billups S Neville M Rudolph M Porter W Schedin P Identifying significant temporal variation in time course microarray data without replicates BMC Bioinformatics 2009 10 96 http://www.biomedcentral.com/1471-2105/10/96 19323838 10.1186/1471-2105-10-96 PMC2682797

[B21] Wu S Wu H More powerful significant testing for time course gene expression data using functional principal component analysis approaches BMC Bioinformatics 2013 14 6 http://www.biomedcentral.com/1471-2105/14/6 23323795 10.1186/1471-2105-14-6 PMC3617096

[B22] Goeman JJ Buhlmann P Analyzing gene expression data in terms of gene sets: methodological issues Bioinformatics 2007 23 8 980 987 17303618 10.1093/bioinformatics/btm051

[B23] Dinu I Potter JD Mueller T Liu Q Adewale AJ Jhangri GS Einecke G Famulski KS Halloran P Yasui Y Gene-set analysis and reduction Brief Bioinform 2008 10 24 34 18836208 10.1093/bib/bbn042 PMC2638622

[B24] Draghici S Khatri P Tarca AL Amin K Done A Voichita C Georgescu C Romero R A systems biology approach for pathway level analysis Genome Research 2007 17 10 1537 1545 17785539 10.1101/gr.6202607 PMC1987343

[B25] Tarca AL Draghici S Khatri P Hassan SS Mittal P Kim Js Kim CJ Kusanovic JP Romero R A novel signaling pathway impact analysis Bioinformatics 2009 25 75 82 18990722 10.1093/bioinformatics/btn577 PMC2732297

[B26] Hosack D Dennis G Sherman B Lane H Lempicki R Identifying biological themes within lists of genes with EASE Genome Biol 2003 4 R70 14519205 10.1186/gb-2003-4-10-r70 PMC328459

[B27] Khatri P Draghici S Ontological analysis of gene expression data: current tools, limitations, and open problems Bioinformatics 2005 21 3587 3595 15994189 10.1093/bioinformatics/bti565 PMC2435250

[B28] Vencio R Shmulevich I ProbCD: enrichment analysis accounting for categorization uncertainty BMC Bioinformatics 2007 8 383 17935624 10.1186/1471-2105-8-383 PMC2169266

[B29] Emmert-Streib F The Chronic Fatigue Syndrome: A Comparative Pathway Analysis Journal of Computational Biology 2007 14 7 961 972 17803373 10.1089/cmb.2007.0041

[B30] Subramanian A Tamayo P Mootha VK Mukherjee S Ebert BL Gillette MA Paulovich A Pomeroy SL Golub TR Lander ES Mesirov JP Gene set enrichment analysis: A knowledge-based approach for interpreting genome-wide expression profiles Proceedings of the National Academy of Sciences of the United States of America 2005 102 43 15545 15550 16199517 10.1073/pnas.0506580102 PMC1239896

[B31] Mansmann U Meister R Testing Differential Gene Expression in Functional Groups. Goeman's Global Test versus an ANCOVA Approach Methods of Inf Med 2005 44 449 53 16113772

[B32] Tsai CA Chen JJ Multivariate analysis of variance test for gene set analysis Bioinformatics 2009 25 897 903 19254923 10.1093/bioinformatics/btp098

[B33] Dinu I Potter J Mueller T Liu Q Adewale A Jhangri G Einecke G Famulski K Halloran P Yasui Y Improving gene set analysis of microarray data by SAM-GS BMC Bioinformatics 2007 8 242 17612399 10.1186/1471-2105-8-242 PMC1931607

[B34] Tian L Greenberg SA Kong SW Altschuler J Kohane IS Park PJ Discovering statistically significant pathways in expression profiling studies Proceedings of the National Academy of Sciences of the United States of America 2005 102 38 13544 13549 16174746 10.1073/pnas.0506577102 PMC1200092

[B35] Massa MS Chiogna M Romualdi C Gene set analysis exploiting the topology of a pathway BMC Systems Biology 2010 4 121 20809931 10.1186/1752-0509-4-121 PMC2945950

[B36] Martini P Sales G Massa MS Chiogna M Romualdi C Along signal paths: an empirical gene set approach exploiting pathway topology Nucleic Acids Research 2013 41 e19 http://nar.oxfordjournals.org/content/41/1/e19.abstract 23002139 10.1093/nar/gks866 PMC3592432

[B37] Goeman JJ van de Geer SA de Kort F van Houwelingen HC A global test for groups of genes: testing association with a clinical outcome Bioinformatics 2004 20 93 99 http://bioinformatics.oxfordjournals.org/content/20/1/93.abstract 14693814 10.1093/bioinformatics/btg382

[B38] Ackermann M Strimmer K A general modular framework for gene set enrichment analysis BMC Bioinformatics 2009 10 47 http://www.biomedcentral.com/1471-2105/10/47 19192285 10.1186/1471-2105-10-47 PMC2661051

[B39] Liu Q Dinu I Adewale A Potter J Yasui Y Comparative evaluation of gene-set analysis methods BMC Bioinformatics 2007 8 431 17988400 10.1186/1471-2105-8-431 PMC2238724

[B40] Nam D Kim SY Gene-set approach for expression pattern analysis Brief Bioinform 2008 9 189 197 18202032 10.1093/bib/bbn001

[B41] Laurent J Pierre N Dudoit S Gains in Power from Structured Two-Sample Tests of Means on Graphs Annals of Applied Statistics 2012 in press

[B42] Antonov AV Schmidt EE Dietmann S Krestyaninova M Hermjakob H R spider: a network-based analysis of gene lists by combining signaling and metabolic pathways from Reactome and KEGG databases Nucleic Acids Research 2010 38 suppl 2 W78 W83 20519200 10.1093/nar/gkq482 PMC2896180

[B43] Isci S Ozturk C Jones J Otu HH Pathway analysis of high-throughput biological data within a Bayesian network framework Bioinformatics 2011 27 12 1667 1674 21551144 10.1093/bioinformatics/btr269

[B44] Sales G Calura E Cavalieri D Romualdi C graphite - a Bioconductor package to convert pathway topology to gene network BMC Bioinformatics 2012 13 20 22292714 10.1186/1471-2105-13-20 PMC3296647

[B45] Sales G Calura E Martini P Romualdi C Graphite Web: web tool for gene set analysis exploiting pathway topology Nucleic Acids Research 2013 http://nar.oxfordjournals.org/content/early/2013/05/10/nar.gkt386.abstract 10.1093/nar/gkt386 PMC3977659 23666626

[B46] Zhao P Iezzi S Carver E Dressman D Gridley T Sartorelli V Hoffman EP Slug Is a Novel Downstream Target of MyoD: TEMPORAL PROFILING IN MUSCLE REGENERATION Journal of Biological Chemistry 2002 277 33 30091 30101 http://www.jbc.org/content/277/33/30091.abstract 12023284 10.1074/jbc.M202668200

[B47] Lauritzen SL Graphical models 1996 Clarendon Press, Oxford

[B48] Dai M Wang P Boyd AD Kostov G Athey B Jones EG Bunney WE Myers RM Speed TP Akil H Watson SJ Meng F Evolving gene/transcript definitions significantly alter the interpretation of GeneChip data Nucleic Acids Research 2005 33 20 e175 http://nar.oxfordjournals.org/content/33/20/e175.abstract 16284200 10.1093/nar/gni179 PMC1283542

[B49] Smoot ME Ono K Ruscheinski J Wang PL Ideker T Cytoscape 2.8: new features for data integration and network visualization Bioinformatics 2011 27 3 431 432 21149340 10.1093/bioinformatics/btq675 PMC3031041

[B50] Tidball JG Villalta SA Regulatory interactions between muscle and the immune system during muscle regeneration American Journal of Physiology-Regulatory, Integrative and Comparative Physiology 2010 298 5 R1173 R1187 10.1152/ajpregu.00735.2009 PMC2867520 20219869

[B51] Sancak Y Peterson TR Shaul YD Lindquist RA Thoreen CC Bar-Peled L Sabatini DM The Rag GTPases bind raptor and mediate amino acid signaling to mTORC1 Science 2008 320 5882 1496 1501 18497260 10.1126/science.1157535 PMC2475333

[B52] Sesodia S Choksi RM Nemeth PM Nerve-dependent recovery of metabolic pathways in regenerating soleus muscles Journal of Muscle Research & Cell Motility 1994 15 5 573 581 7860705 10.1007/BF00121163

[B53] Scheerer N Dehne N Stockmann C Swoboda S Baba HA Neugebauer A Johnson RS Fandrey J Myeloid Hypoxia-Inducible Factor-1*α *Is Essential for Skeletal Muscle Regeneration in Mice The Journal of Immunology 2013 191 407 414 23729446 10.4049/jimmunol.1103779 PMC6614040

[B54] Richard-Bulteau H Serrurier B Crassous B Banzet S Peinnequin A Bigard X Koulmann N Recovery of skeletal muscle mass after extensive injury: positive effects of increased contractile activity American Journal of Physiology-Cell Physiology 2008 294 2 C467 C476 18077604 10.1152/ajpcell.00355.2007

[B55] Dickinson JM Fry CS Drummond MJ Gundermann DM Walker DK Glynn EL Timmerman KL Dhanani S Volpi E Rasmussen BB Mammalian target of rapamycin complex 1 activation is required for the stimulation of human skeletal muscle protein synthesis by essential amino acids The Journal of nutrition 2011 141 5 856 862 21430254 10.3945/jn.111.139485 PMC3077888

