# Supplementary material for: timeClip: pathway analysis for time course data without replicates
Source: BMC Bioinformatics. 2014 May 6;15(Suppl 5):S3. doi: 10.1186/1471-2105-15-S5-S3 (PMC4095003; doi:10.1186/1471-2105-15-S5-S3)
Supplement: Additional file 1 — Additional tables. This file contains additional tables mention on the text (pdf format). [file 1471-2105-15-S5-S3-S1.pdf]

timeClip: pathway analysis for time course data without replicates
Supplementary Information

Paolo Martini, Gabriele Sales, Enrica Calura, Stefano Cagnin, Monica Chiogna
Chiara Romualdi[1]

[1]To whom correspondence should be addressed. Tel: +39 049 8277401; Fax: +39 049 8276159; Email: chiara.romualdi@unipd.it

Additional tables

Additional Table S1 — False positives rate with different pathway dimensions n and regularly sampled time points t.

	$t = 5$	$t = 10$	$t = 15$	$t = 20$	$t = 25$	$t = 30$
$n = 5$	0.19	0.11	0.07	0.05	0.06	0.05
$n = 10$	0.19	0.09	0.08	0.08	0.08	0.06
$n = 15$	0.21	0.09	0.07	0.07	0.06	0.06
$n = 20$	0.21	0.09	0.07	0.07	0.06	0.06
$n = 25$	0.19	0.11	0.07	0.05	0.06	0.07
$n = 30$	0.20	0.10	0.07	0.07	0.08	0.09

Additional Table S2 — Power estimate in case of $n = 30$ and different time course length t and time dependent genes s. Regularly sampled time points.

	$s = 3$	$s = 6$	$s = 9$	$s = 15$	$s = 21$	$s = 30$
$t = 5$	0.27	0.26	0.26	0.26	0.30	0.26
$t = 10$	0.56	0.53	0.51	0.52	0.51	0.50
$t = 15$	0.75	0.72	0.74	0.73	0.72	0.72
$t = 20$	0.86	0.83	0.83	0.84	0.83	0.82
$t = 25$	0.90	0.90	0.91	0.87	0.90	0.85
$t = 30$	0.91	0.92	0.92	0.91	0.91	0.90
