# Supplementary figures and images for: timeClip: pathway analysis for time course data without replicates
Source: BMC Bioinformatics. 2014 May 6;15(Suppl 5):S3. doi: 10.1186/1471-2105-15-S5-S3 (PMC4095003; doi:10.1186/1471-2105-15-S5-S3)

# HIF-1 SIGNALING PATHWAY

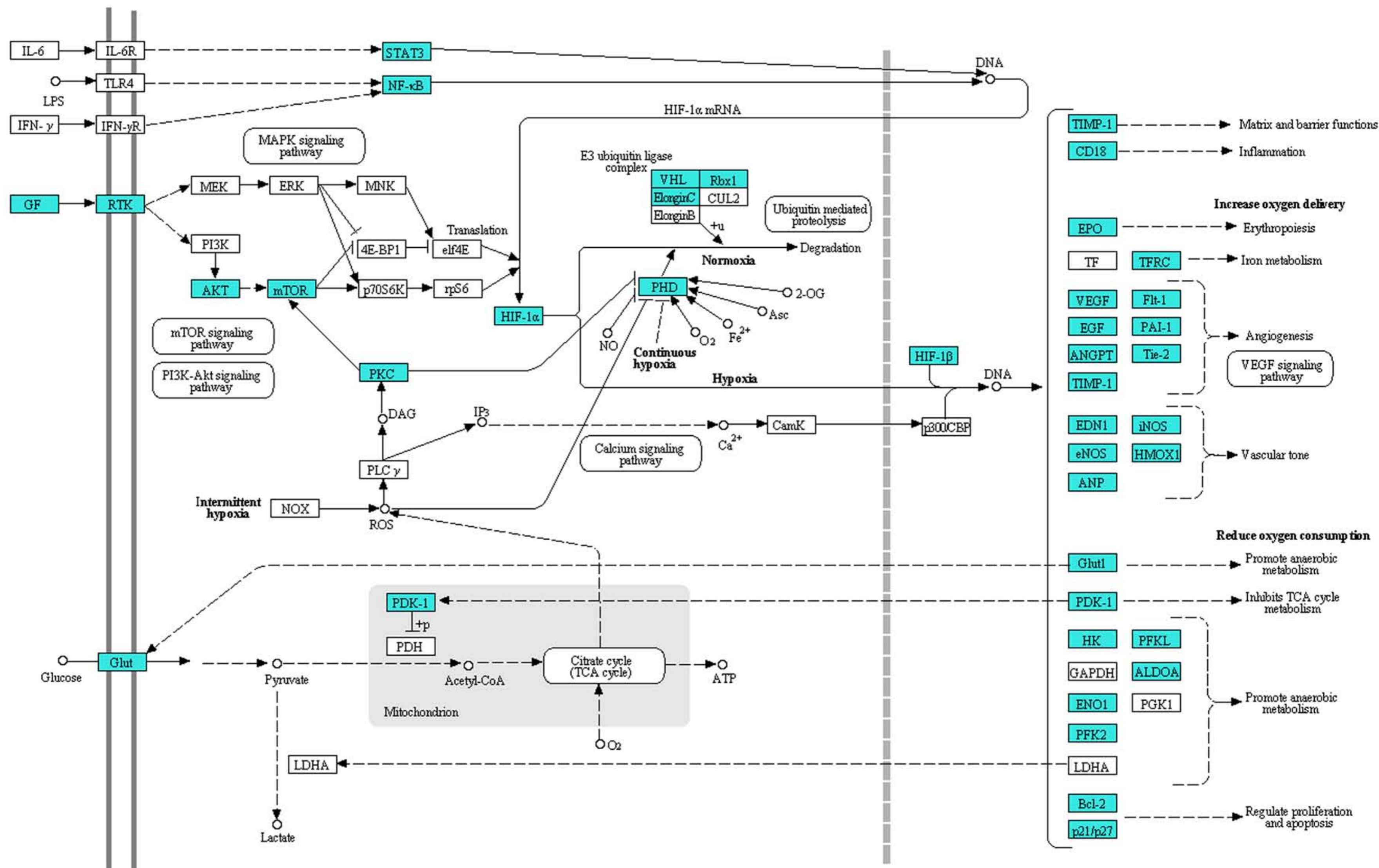

Supplement: Additional file 3 — Activation of the HIF-1 signaling pathway. KEGG representation of HIF-1 signaling pathway. Genes of the 37 clique long path colored in cyan. [file 1471-2105-15-S5-S3-S3.pdf]
